# Genome-wide interaction analysis identified low-frequency variants with sex disparity in lung cancer risk

**DOI:** 10.1093/hmg/ddac030

**Published:** 2022-02-09

**Authors:** Yafang Li, Xiangjun Xiao, Jianrong Li, Jinyoung Byun, Chao Cheng, Yohan Bossé, James McKay, Demetrios Albanes, Stephen Lam, Adonina Tardon, Chu Chen, Stig E Bojesen, Maria T Landi, Mattias Johansson, Angela Risch, Heike Bickeböller, H-Erich Wichmann, David C Christiani, Gad Rennert, Susanne Arnold, Gary Goodman, John K Field, Michael P A Davies, Sanjay S Shete, Loic Le Marchand, Olle Melander, Hans Brunnström, Geoffrey Liu, Rayjean J Hung, Angeline S Andrew, Lambertus A Kiemeney, Hongbing Shen, Ryan Sun, Shan Zienolddiny, Kjell Grankvist, Mikael Johansson, Neil Caporaso, Dawn M Teare, Yun-Chul Hong, Philip Lazarus, Matthew B Schabath, Melinda C Aldrich, Ann G Schwartz, Ivan Gorlov, Kristen Purrington, Ping Yang, Yanhong Liu, Younghun Han, Joan E Bailey-Wilson, Susan M Pinney, Diptasri Mandal, James C Willey, Colette Gaba, Paul Brennan, Christopher I Amos, INTEGRAL-ILCCO lung cancer consortium

**Affiliations:** Institute for Clinical and Translational Research, Baylor College of Medicine, Houston, TX 77030, USA; Section of Epidemiology and Population Sciences, Department of Medicine, Baylor College of Medicine, Houston, TX 77030, USA; Dan L Duncan Comprehensive Cancer Center, Baylor College of Medicine, Houston, TX 77030, USA; Institute for Clinical and Translational Research, Baylor College of Medicine, Houston, TX 77030, USA; Institute for Clinical and Translational Research, Baylor College of Medicine, Houston, TX 77030, USA; Institute for Clinical and Translational Research, Baylor College of Medicine, Houston, TX 77030, USA; Section of Epidemiology and Population Sciences, Department of Medicine, Baylor College of Medicine, Houston, TX 77030, USA; Institute for Clinical and Translational Research, Baylor College of Medicine, Houston, TX 77030, USA; Section of Epidemiology and Population Sciences, Department of Medicine, Baylor College of Medicine, Houston, TX 77030, USA; Dan L Duncan Comprehensive Cancer Center, Baylor College of Medicine, Houston, TX 77030, USA; Institut universitaire de cardiologie et de pneumologie de Québec, Department of Molecular Medicine, Laval University, Quebec City G1V 4G5, Canada; Section of Genetics, International Agency for Research on Cancer, World Health Organization, Lyon 69372, France; Division of Cancer Epidemiology and Genetics, National Cancer Institute, National Institutes of Health, Bethesda, MD 20850, USA; Department of Integrative Oncology, University of British Columbia, Vancouver, BC V5Z 1L3, Canada; Public Health Department, University of Oviedo, ISPA and CIBERESP, Asturias 33003, Spain; Program in Epidemiology, Public Health Sciences Division, Fred Hutchinson Cancer Research Center, Seattle, WA 98109, USA; Department of Clinical Biochemistry, Copenhagen University Hospital, Copenhagen 2600, Denmark; Faculty of Health and Medical Sciences, University of Copenhagen, Copenhagen 2177, Denmark; Division of Cancer Epidemiology and Genetics, National Cancer Institute, National Institutes of Health, Bethesda, MD 20850, USA; Section of Genetics, International Agency for Research on Cancer, World Health Organization, Lyon 69372, France; Thoraxklinik at University Hospital Heidelberg, Heidelberg 69126, Germany; Translational Lung Research Center Heidelberg (TLRC-H), Heidelberg 69120, Germany; University of Salzburg and Cancer Cluster Salzburg, 5020, Austria; Department of Genetic Epidemiology, University Medical Center, Georg-August-University Göttingen, 37099, Germany; Institute of Medical Statistics and Epidemiology, Technical University Munich, 80333, Germany; Departments of Environmental Health and Epidemiology, Harvard TH Chan School of Public Health, Boston, MA 02115, USA; Clalit National Cancer Control Center at Carmel Medical Center and Technion Faculty of Medicine, Haifa 3436212, Israel; University of Kentucky, Markey Cancer Center, Lexington, Kentucky 40536, USA; Swedish Cancer Institute, Seattle, WA 98104, USA; Institute of Translational Medicine, University of Liverpool, Liverpool L69 7BE, United Kingdom; Institute of Translational Medicine, University of Liverpool, Liverpool L69 7BE, United Kingdom; Department of Biostatistics, The University of Texas, M.D. Anderson Cancer Center, Houston, TX 77030, USA; Department of Epidemiology, The University of Texas MD Anderson Cancer Center, Houston, TX 77030, USA; Epidemiology Program, University of Hawaii Cancer Center, Honolulu, HI 96813, USA; Faculty of Medicine, Lund University, Lund 22184, Sweden; Faculty of Medicine, Lund University, Lund 22184, Sweden; University Health Network- The Princess Margaret Cancer Centre, Toronto, CA ON, M5G 2C1, Canada; Luenfeld-Tanenbaum Research Institute, Sinai Health System, Toronto ON, M5G 1X5, Canada; Division of Epidemiology, Dalla Lana School of Public Health, University of Toronto, Toronto ON, M5T 3M7, Canada; Departments of Epidemiology and Community and Family Medicine, Dartmouth College, Hanover, NH 03755, USA; Radboud University Medical Center, Nijmegen 6525, The Netherlands; Department of Epidemiology and Biostatistics, Jiangsu Key Lab of Cancer Biomarkers, Prevention and Treatment, Collaborative Innovation Center for Cancer Personalized Medicine, School of Public Health, Nanjing Medical University, Nanjing 211166, P.R. China; Department of Biostatistics, The University of Texas, M.D. Anderson Cancer Center, Houston, TX 77030, USA; National Institute of Occupational Health, Oslo 0304, Norway; Department of Medical Biosciences, Umeå University, Umeå 901 87, Sweden; Department of Radiation Sciences, Umeå University, Umeå 901 87, Sweden; Division of Cancer Epidemiology and Genetics, National Cancer Institute, National Institutes of Health, Bethesda, MD 20850, USA; Population Health Sciences Institute, Newcastle University, Newcastle upon Tyne, NE2 4AX, UK; Department of Preventive Medicine, Seoul National University College of Medicine, Seoul 03080, Republic of Korea; Department of Pharmaceutical Sciences, College of Pharmacy, Washington State University, Spokane, Washington 99202, USA; Department of Cancer Epidemiology, H. Lee Moffitt Cancer Center and Research Institute, Tampa, FL 33612, USA; Department of Thoracic Surgery, Division of Epidemiology, Vanderbilt University Medical Center Nashville, TN 37232, USA; Department of Oncology, Wayne State University School of Medicine, Detroit, MI 48201, USA; Karmanos Cancer Institute, Detroit, MI 48201, USA; Institute for Clinical and Translational Research, Baylor College of Medicine, Houston, TX 77030, USA; Section of Epidemiology and Population Sciences, Department of Medicine, Baylor College of Medicine, Houston, TX 77030, USA; Dan L Duncan Comprehensive Cancer Center, Baylor College of Medicine, Houston, TX 77030, USA; Karmanos Cancer Institute, Detroit, MI 48201, USA; Division of Epidemiology, Department of Health Sciences Research, Mayo Clinics Rochester, MN, 55905, USA; Section of Epidemiology and Population Sciences, Department of Medicine, Baylor College of Medicine, Houston, TX 77030, USA; Dan L Duncan Comprehensive Cancer Center, Baylor College of Medicine, Houston, TX 77030, USA; Institute for Clinical and Translational Research, Baylor College of Medicine, Houston, TX 77030, USA; Section of Epidemiology and Population Sciences, Department of Medicine, Baylor College of Medicine, Houston, TX 77030, USA; National Human Genome Research Institute, NIH, Baltimore, MD 20892, USA; University of Cincinnati College of Medicine, Cincinnati, OH 45267, USA; Louisiana State University Health Sciences Center, New Orleans, LA 70112, USA; College of Medicine and Life Sciences, University of Toledo, Toledo, OH 43614, USA; The University of Toledo College of Medicine, Toledo, OH 43614, USA; Section of Genetics, International Agency for Research on Cancer, World Health Organization, Lyon 69372, France; Institute for Clinical and Translational Research, Baylor College of Medicine, Houston, TX 77030, USA; Section of Epidemiology and Population Sciences, Department of Medicine, Baylor College of Medicine, Houston, TX 77030, USA; Dan L Duncan Comprehensive Cancer Center, Baylor College of Medicine, Houston, TX 77030, USA

## Abstract

Differences by sex in lung cancer incidence and mortality have been reported which cannot be fully explained by sex differences in smoking behavior, implying existence of genetic and molecular basis for sex disparity in lung cancer development. However, the information about sex dimorphism in lung cancer risk is quite limited despite the great success in lung cancer association studies. By adopting a stringent two-stage analysis strategy, we performed a genome-wide gene–sex interaction analysis using genotypes from a lung cancer cohort including ~ 47 000 individuals with European ancestry. Three low-frequency variants (minor allele frequency < 0.05), rs17662871 [odds ratio (OR) = 0.71, *P* = 4.29×10^−8^); rs79942605 (OR = 2.17, *P* = 2.81×10^−8^) and rs208908 (OR = 0.70, *P* = 4.54×10^−8^) were identified with different risk effect of lung cancer between men and women. Further expression quantitative trait loci and functional annotation analysis suggested rs208908 affects lung cancer risk through differential regulation of Coxsackie virus and adenovirus receptor gene expression in lung tissues between men and women. Our study is one of the first studies to provide novel insights about the genetic and molecular basis for sex disparity in lung cancer development.

## Introduction

Lung cancer is the leading cause of cancer death for both men and women worldwide with a complex genetic and molecular mechanism. Differences by sex in lung cancer incidence and mortality have been reported (1,2). Historically, the sex difference in lung cancer risk development was explained by sex-differences in smoking behavior as women were less likely to smoke cigarettes, initiated smoking at older ages and smoked fewer cigarettes per day (3,4). In 2018, a study on incidence rate of lung cancer between men and women, on the basis of data including 300 343 cases of lung cancer in non-Hispanic Whites, showed that the female-to-male incidence rate ratio had increased over the past two decades, whereas the prevalence of smoking among women had been approaching to the prevalence among men since 1965 (5). And the ratio exceeded 1 in the age groups of 30–34, 35–39, 40–44 and 45–49 years in people with European ancestry. For example, the female-to-male incidence rate ratio among individuals between 40 and 44 years of age had increased from 0.88 [95% CI (confidence interval): (0.84, 0.92)] during the 1995–1999 period to 1.17 [95% CI: (1.11, 1.23)] during the 2010–2014 period and from 0.81 [95% CI: (0.78, 0.83) to 1.13 [95% CI: (1.09, 1.16)]. A similar trend was also identified in Asian or pacific islander and Hispanic population. As smoking behavior has become increasingly similar between men and women, there is growing evidence that sex differences in lung cancer risk cannot be fully explained by differences in smoking behavior, implying sex-based variations in the genetic and molecular basis for lung cancer (5,6). However, the information for sex differences in lung cancer risk remains poorly understood despite the extensive efforts spent in lung cancer research and great success in identifying genetic factors through lung cancer association studies.

Genome-wide association studies (GWAS) have been used to identify sexual dimorphism in genetic susceptibility to lung cancer. Sex-specific GWAS identified *VTI1A, ACVR1B* and *FOXP4-AS1* genes in women that influence lung cancer development (7,8). However, prior female sex-specific GWAS could not test for statistical significance in males or for gene–sex interactions. Gene–sex interaction analysis, on the other hand, will evaluate the information from both male and female group systematically and can identify the variants with significant difference between men and women, although those variants may not achieve genome-wide significance in stratified analysis. But genome-wide genetic interaction (GWGI) studies still remain challenging as most GWAS were designed for main effect detection and have had limited power for interaction analysis. Analytical studies have shown that a sample size that is at least 4-fold larger is required for detecting significant effects in interaction analysis using a standard case–control design compared with detecting significant main effects. An even larger sample size is required when the effect size is modest or the risk allele has a lower frequency (9). A case-only approach has been shown to be much more powerful in detection of an interaction effect than a standard case–control design in the absence of gene–environment correlation (10–14). However, the test validity is destroyed if the gene–environment independence assumption is violated. Researchers proposed a combined case-only and case–control approach in GWGI: step 1, a case-only analysis to test the association between Single nucleotide polymorphism (SNPs) and environmental/or biological factors; step 2, candidate SNPs from step 1 are further evaluated using standard case–control logistic interaction analysis (15–17). This two-step study design benefits from both increased power from case-only analysis and robustness to gene–environment independence assumption. Researchers have applied this approach in gene–environment interaction analysis and identified several novel variants in various human disorders including lung cancer (17–19). In this report, we applied this two-step approach on genome-wide gene–sex interaction analysis in lung cancer, aiming to identify novel susceptibility loci with different or inverse effects between male and female population that are not significant in main-effect association analysis.

Functional inference of genetic variants is important for gaining insights about the molecular mechanism of the disease and clinical application of GWAS findings. Over 90% of GWAS variants are located within non-coding regions and they may affect disease risk through regulating expression of nearby genes (20,21). Expression quantitative trait loci (eQTL) analysis, an allelic association analysis with gene expression, provides a straightforward method to identify susceptibility genes associated with the GWAS hits and it has been widely used in GWAS to investigate the regulatory effect of variants (22–24). However, there are few or no reports about the application of eQTL in gene–sex interaction analysis in human diseases because of the limited studies in sexual dimorphism in disease risk. Another important approach for assessment of genetic variants is functional annotation analysis. Researchers developed various tools to infer the functional roles for the variants such as combined annotation dependent depletion (CADD) and RegulomeDB (25–27). These tools provide insightful information about the functional inference and are very helpful for fine mapping and pinning down the true causal allele.

In this report, we performed a genome-wide gene–sex interaction analysis using genotype from a large lung cancer cohort including ~ 47 000 individuals with European ancestry. Adopting the two-step analysis strategy, we identified novel variants with different risk effects between men and women that were missed by main-effect association analysis. eQTL and functional annotation analysis provided further information about the functional role for the identified genetic variants and supplied multiple lines of evidence for the sexual disparity in lung cancer development. Our study is one of the first studies for sexual difference in risk of cancer development between men and women. It is also the largest scanning for gene–sex interaction in lung cancer and explored the genetic and molecular basis for sexual differences in risk of this deadly disease.

## Results

### Novel signals from genome-wide gene–sex interaction analysis

In the case-only analysis stage, a total of 19 943 415 and 10 359 674 SNPs were tested in the discovery and replication studies, respectively. Figure 2a displays the Manhattan plot of joint case-only analysis of gene–sex interactions in lung cancer. No inflated type I error rate was detected (lambda = 0.98). There were eight SNPs with a case-only joint *P*-value <5×10^−8^ and joint *P*-values were more significant than those from both discovery and replication studies. Those SNPs were submitted to further case–control interaction analysis using pooled discovery and replication data including 24 223 lung cancer patients and 22 560 controls. Three of these had interaction *P*-values < 0.05 in case–control analysis (Supplementary Material, Table S2). All three candidate variants had minor allele frequency (MAF) < 0.05 and thus were further evaluated using Firth logistic regression method. The three variants remained significant in Firth analysis and the results are reported in Table 2.

SNP rs17662871, located near the carbonic anhydrase 10 (*CA10*) gene, had an OR of 0.69 and case-only *P*-value of 1.21×10^−7^ in discovery study, and OR of 0.80 and *P*-value of 7.03×10^−2^ in the replication study. Further case–control validation using Firth test detected an interaction OR of 0.78 and *P*-value of 4.06×10^−3^. SNP rs208908, located near Coxsackie virus and adenovirus receptor (*CXADR*) gene, had a *P*-value of 2.84×10^−6^ (OR = 0.71) and 4.49×10^−3^ (OR = 0.67) in case-only discovery and replication studies, respectively. It has a meta-analysis *P*-value of 4.54×10^−8^ (OR = 0.70) and *P*-value of 1.74×10^−2^ (OR = 0.80) in case–control analysis. rs79942605 had an OR of 2.17 (*P* = 2.81×10^−8^) in joint case-only analysis. In addition, case–control analysis identified an interaction OR of 1.68 (*P* = 1.64×10^−2^) in lung cancer cohort. We performed the same test in other lung cancer subtypes, such as lung adenocarcinoma and squamous lung cancer, etc. The signals for the top 10 variants in case-only analysis were reported for each lung cancer subtype although no significant variants were identified (Supplementary Material, Table S3).

### Regional plot at the significant regions

We examined the regional information around the significant findings. rs17662871, close to *CA10* gene, was the single variant achieving genome-wide significance of gene–sex interaction in the region and there were no other variants that were in strong linkage disequilibrium (LD) with this variant. This variant had an imputation quality score of 0.72 (Fig. 2b top). SNP rs79942605, located within mono-ADP ribosylhydrolase 2 (*MACROD2*) gene, had a *P*-value of 2.81×10^−8^ and OR 2.17 in joint case-only analysis. Another variant rs76314075, in high LD with rs79942605 (r^2^ ≥0.8), had an OR of 2.11 and *P*-value of 1.33×10^−7^ in the joint analysis (Fig. 2b middle). For the third novel variant, rs208908 (OR = 0.70, *P* = 4.54×10^−8^), located upstream of *CXADR* gene, we found eight variants with joint case-only *P*-value <1×10^−5^ and the strongly associated variant rs9637031 had an OR of 0.70 and *P*-value of 1.34×10^−7^ in the joint analysis (Fig. 2b bottom).

### Stratified analysis of lung cancer risk at significant SNPs

To explore how sex influenced genetic risk in lung cancer, we conducted stratified lung cancer risk analysis in men and women using discovery and replication combined dataset. We observed very distinct lung cancer risk patterns between men and women at the three identified variants. For example, rs208908 had a protective effect for lung cancer in women with OR of 0.81 (*P* = 1.17×10^−3^) but had no significant effect in males (OR = 1.03, *P* = 0.59) (Fig. 2c). Similarly, SNP rs17662871 had a protective effect for lung cancer in women with OR of 0.86 (*P* = 1.44×10^−2^). We further stratified the analysis by smoking status (ever. vs. never smokers) and the risk effect did not vary by smoking status for these two SNPs. rs79942605, on the other hand, had an increased risk for lung cancer in women (OR = 1.52, *P*-value = 4.76×10^−3^). And this variant only had risk effect in ever smoking women (OR = 2.03, *P* = 4.47×10^−4^), whereas not in never smoking women (OR = 0.60, *P* = 1.79×10^−1^).

### eQTL analysis

We conducted eQTL analysis to further evaluate the interaction effect associated with nearby gene expression for each of the candidate SNPs. Unfortunately, for rs79942605, in the *MARCROD2* gene, no SNP data were available in genotype-tissue expression (GTEx). There were also no data for rs76314075 that is in strong LD with rs79942605 (r^2^ > 0.8) in GTEx. rs17662871, located upstream to *CA10* gene, was an imputed SNP in GTEx with allele frequency of 0.05. We applied a best guess algorithm to assign most likely genotypes to each individual to transform the dosage into genotype. Because the variant is uncommon we did not model an additional group homozygous for the rare variant. After filtering the individuals with very low expression [reads per kilobase million (rpkm) < 0.25], 113 men and 37 women were available in the analysis, and only 5 of them were carriers. The gene expression [log2(rpkm)] between genotype ‘0’ and ‘1’ group was compared and the *P*-value was 2.21×10^−3^ in male+female combined group and 4.55×10^−3^ in male group. However, there was no valid test for women group as there was only one individual in genotype ‘1’ group (Fig. 3a left).

rs208908 was also a low-frequency variant with MAF of 0.04. It was a genotyped SNP in GTEx and there were 240 men and 132 women for gene expression association analysis after gene expression filtering. In the male+female combined group, the *CXADR* gene expression level was not significantly different between carrier and non-carrier group (*P* = 0.47). However, this gene had a lower expression in the carrier group compared with the non-carrier group at marginal level in men (*P* = 5.54×10^−2^) and had higher expression in carrier group compared with non-carrier group in women (*P* = 5.47×10^−2^) (Fig. 3a middle). A generalized linear model was applied to test the gene–sex interaction in *CXADR* gene expression prediction and there was a significant interaction effect with *P*-value of 3.85×10^−3^ (Supplementary Material, Table S4). We further expand the eQTL analysis to 229 SNPs located between 18 745 702 and 18 806 105 bp within the candidate region (hg19). The z-score from joint case-only was plotted against the z-score from eQTL analysis for those SNPs (Fig. 3a right). The variants with significant genetic interactions in lung cancer risk prediction also had significant interactions in gene expression prediction, and those variants were in strong LD with candidate SNP rs208908 (r^2^ > 0.8). These integrated results from genetic association and eQTL analysis suggested rs208908 had different risk effect in lung cancer between men and women through *CXADR* gene expression regulation in lung tissues.

### Functional annotation analysis

We retrieved the SNPs with case-only meta-analysis *P*-value <1×10^−5^ from the three significant regions and queried the functional inference for each of the variants. Twelve SNPs were submitted to the analysis. rs79942605, the significant variant located in *MACROD2*, had the largest scaled-CADD (PHRED) score of 8.2 indicating top 15% of all reference genome of it being a functional allele. Among the nine SNPs from upstream of *CXADR* gene, rs208908 was predicted to be a transcription factor binding site (TBFS) of DNase peak with probability of 0.92 by RegulomeDB program (Table 3). Considering the strong probability of rs208908 being a regulatory SNP in TBFS, we used PROMO, a program for the identification of gene expression regulatory motif such as putative TFBS in DNA sequence, to search for motif located upstream of *CXADR* gene. We identified an 8-bp TBFS motif including six highly conserved variants in the human genome (Fig. 3b upper). The minor allele of rs208908 ‘A’ in the motif is predicted to increase the binding of transcription factor (TF) GR-beta and YY1 at this site. Both of the TFs have been reported involved in tumorigenesis (28,29). RegulomeDB searched the database of annotated SNPs with known and predicted regulatory elements in the intergenic regions of the human genome and found rs208908 being involved in chromatin state with strong transcription activity in human brain, pancreas, embryo tissues, etc. The DNase-seq analysis in HEK293T (Human embryonic kidney) cells also detected a peak covering rs208908 suggesting it was located within a *cis*-regulatory DNA sequence element (RegulomeDB, Fig. 3b bottom).

## Discussion

Sex is an important biological factor in human disease development and extensive studies have been conducted to demonstrate sex differences in incidence, prognosis and treatment of various diseases including cancer (1,2). In 2016, a comprehensive characterization of molecular differences between male and female patients in 13 cancer types using multidimensional genomic data in the Cancer Genome atlas(TCGA) categorized lung cancer into the strong sex effect group with more extensive sex-biased genetic and molecular signatures (30). However, differences in risk on the basis of sex are one of the least studied factors in cancer susceptibility. And study of sex disparity in lung cancer development is quite limited despite the success of GWAS in lung cancer research during the past decade. A comprehensive gene–sex interaction scanning combined with functional annotation analysis provided exploratory insights about molecular mechanism underlying difference in lung cancer risk between men and women.

Leveraging the rich resource from integrative analysis of lung cancer etiology and risk (INTEGRAL)-international lung cancer consortium (ILCCO) lung cancer consortium, we conducted one of the largest gene–sex interaction analysis in overall lung cancer as well as lung cancer subtypes using imputed genotype from ~ 47 000 individuals with European ancestry. By adopting a robust and stringent two-stage analysis strategy, we identified three significant gene–sex interactions in overall lung cancer and all the three variants had small MAF between 0.01 and 0.05. Considering all the three variants had MAF < 0.05 and the standard regression method may not preserve the type I error rate for variants with low allele frequency, we further applied the Firth test and validated the signals at the three SNPs. We even retrieved ~ 200 SNPs from the three candidate regions and compared the results between regular logistic regression method and Firth logistic regression method. The beta estimation and *P*-values between these two methods were highly concordant with each other implying that our results from case-only analysis were reliable (Supplementary Material, Figure S5). Among the three significant variants, rs17662871 and rs208908 displayed a protective effect for lung cancer in women and no effect in men. And the protective effect did not vary much by smoking status (ever vs. never) in women. rs79942605, from *MACROD2* gene, displayed strong risk effect in only ever smoking women (OR = 1.52, *P* = 4.76×10^−3^), whereas no effect in men or never smoking women was observed. We also conducted the analysis in lung adenocarcinoma and squamous lung cancer but did not identify any significant interactions, possibly because of decreased sample sizes in these subgroups. This suggests that genome-wide scanning for interactions between genes and environmental/biological factors still remains a challenge requiring a more powerful analysis strategy.

All three novel variants identified in this study have significant risk effects in only one gender and non-significant effects in the other, and the non-significant effects tend to be in a reverse direction (Fig. 2C). Those sex-specific effects did not achieve genome-wide significance in regular or sex-specific GWAS analysis thus missed in main-effects analysis. Our findings suggest that gene–sex interaction is a good complement to GWAS in detection of loci with effect in only one gender or with inverse effects between genders.

It is possible that there are some SNPs not significant in case-only analysis but with differential magnitude of associations with lung cancer risk between sexes. We extracted the sex-specific association effects from 22 reported significant variants in lung cancer from European population (Supplementary Material, Table S6) (31,32). All of them have risk effects in both genders although some of them do not achieve genome-wide significance in one gender. Seven SNPs have varied risk effects between male and female groups (|ORmale-ORfemale| > 0.05). The largest variation was from rs9865715 with OR of 1.77 (*P* = 5.42×10^–^8) in women and OR of 1.49 (*P* = 2.38×10^–4^) in men. None of the seven variants had significant results in case-only analysis. These results suggested that the variants with differential magnitude of association in same direction between genders are usually identified in main-effect analysis using regular GWAS or sex-specific GWAS if their main-effect is very significant in one sex. And the gene–sex interactions may impose very negligible variations in risk effects in lung cancer development for those variants.

eQTL analysis has been very useful in GWAS to provide additional functional evidence for the identified susceptibility loci. However, very few studies were reported about its application in interaction analysis between genes and environmental/biological factors probably because of the limited studies in interaction scanning, restricting the contribution of eQTL in genetic association studies. We explored the application of eQTL in gene–sex interaction study. Take variant rs208908 and *CXADR* as an example, we did not identify significant association between rs208908 and *CXADR* gene in main-effect eQTL analysis (*P* = 0.47, Fig. 3a middle). However, we identified distinct gene expression patterns between men and women and we detected significant gene–sex interaction in *CXADR* gene expression prediction (*P* = 3.85×10^−3^). In addition, the signals from gene–sex interactions between lung cancer risk prediction and *CXADR* gene expression prediction are highly correlated among the SNPs in high LD with rs208908 (r^2^ > 0.8) (Fig. 3a right). The eQTL analysis provided strong evidence suggesting rs208908 is a functional variant with difference in lung cancer risk by sex and revealed great potential for application of eQTL in large-scale scanning for interactions between genes and environmental/biological factors.

**Table 1:** Characteristics of participants that were studied during the discovery and replication phases

	Discovery^a^			Replication^b^		
	Male (%)	Female (%)	Total	Male (%)	Female (%)	Total
Controls	7646 (58.6)	5411 (41.4)	13 057	5796 (61.0)	3707 (39.0)	9503
Cases	10 172 (60.4)	6673 (39.6)	16 845	4719 (64.0)	2659 (36.0)	7378
Ever smokers	15 584 (63.9)	8813 (36.1)	24 397	9230 (67.0)	4553 (33.0)	13 783
Never smokers	2234 (40.6)	3271 (59.4)	5505	1285 (41.5)	1813 (58.5)	3098

Samples with European ancestry in INTEGRAL-ILCCO consortium were analyzed in the study. Number of overall lung cancer cases was provided in the table.

^a^Genotype data from Oncoarray study were used in discovery study.

^b^Genotype from the other eight studies were used in replication study.

**Figure 1:**
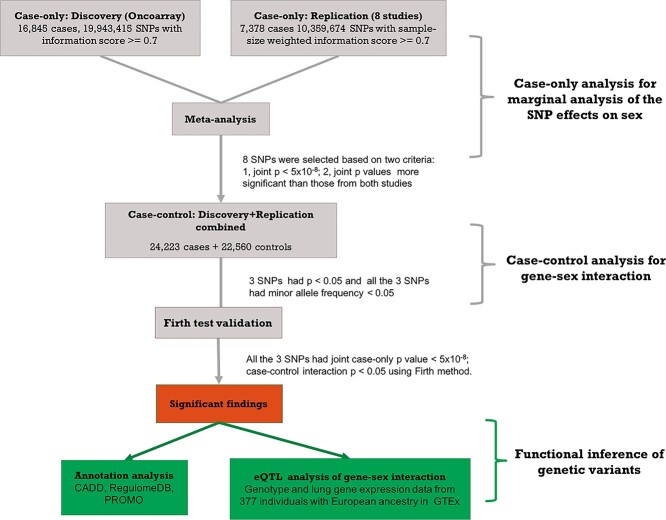
Flow chart of analysis strategy in genome-wide gene–sex interaction analysis in lung cancer. In genetic association analysis, all the tested SNPs have information score ≥ 0.7 in discovery data; information score > 0.2 in each of the eight studies and sample-size weighted information score ≥ 0.7 in replication data. Smoking status (ever vs. never smokers) and first five principal components were adjusted in case-only, case–control and firth validation analysis.

**Figure 2:**
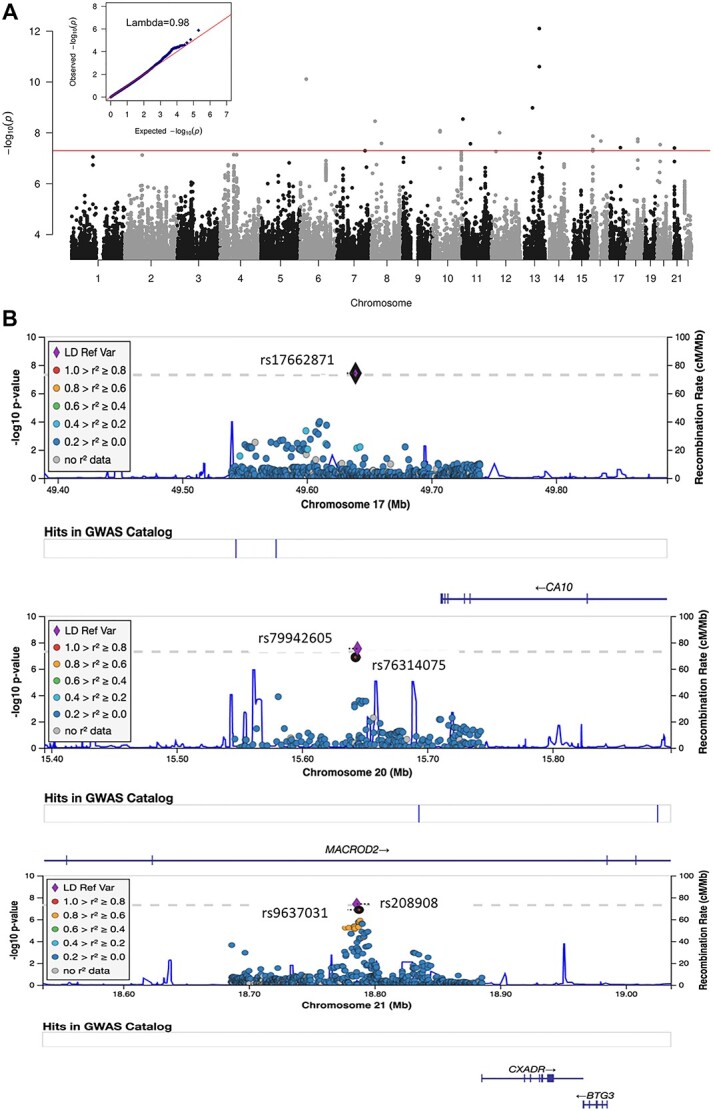

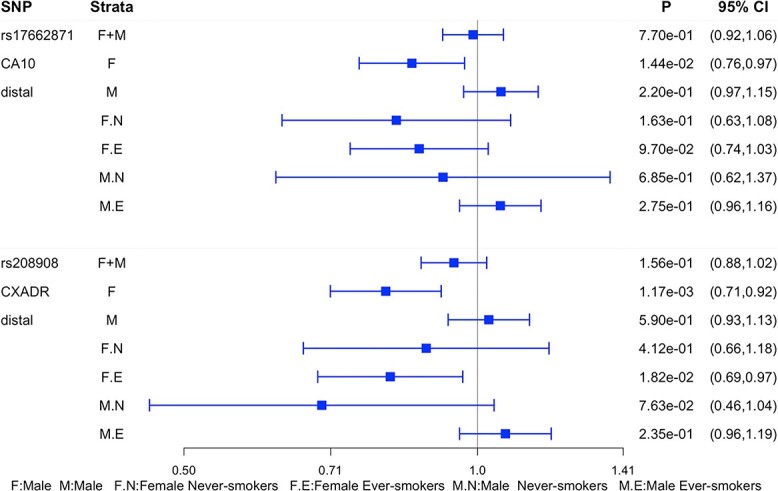
Plot of signals from genome-wide gene–sex interaction analysis in lung cancer. (**a**) Manhattan plot of *P*-values from case-only meta-analysis. No inflated type I error was detected in the analysis (lambda = 0.98). (**b**) Regional plot at three significant regions. (**c**) Forest plot of stratified lung cancer risk for the most significant SNP from each region.

**Figure 3:**
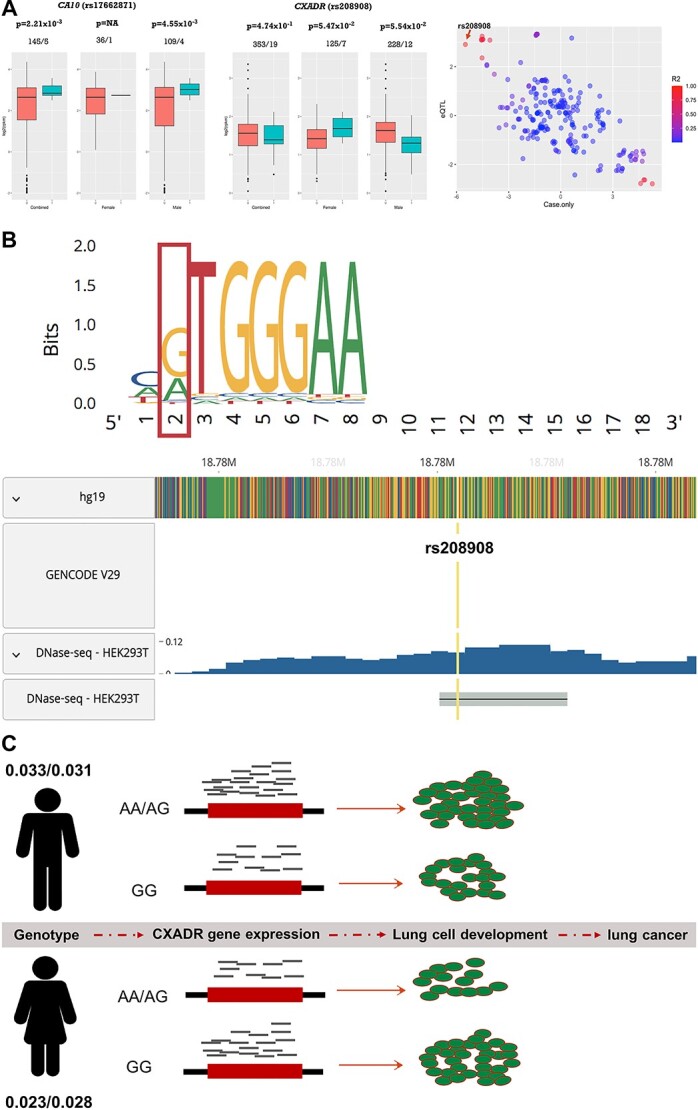
Functional analysis of identified variants. (**a**) eQTL analysis at candidate variants. Left and middle, box plot of gene expression from lung between individuals with none (0) and at least on risk allele (1) in female and male combined, female and male groups for *CA10* and *CXADR*, respectively. The data from samples with Caucasian ancestry in GTEx were used in the analysis. Individuals with rpkm < 0.25 were removed from the analysis. *P*-values and number of samples were labeled above each plot. rs208908 was genotyped in GTEx; rs17662871 was imputed in SNP and SNP dosage ≤0.4 was coded as 0; SNP dosage ≥ 0.6 was coded as 1. Linear regression analysis of gene–sex interaction in predicting *CXADR* gene expression displayed *P* = 3.85×10^−3^. Right, scatter plot of z-scores from 229 SNPs, ranging from 18 745 702 to 18 806 105 (hg19) on chromosome 21 was displayed on the plot. *X*-axis represented gene–sex interaction analysis in lung cancer risk; and *y*-axis represented the z-scores from gene–sex interaction analysis in *CXADR* gene expression prediction. LD r^2^ was computed for each of the SNPs with rs208908 as reference. (**b**) Functional inference of rs208908. The predicted binding motif at rs208908 (highlighted in red color) for TF using PROMO (Upper). DNase-seq analysis in HEK293T (Human embryonic kidney) cells detected a peak covering rs208908 suggesting it was located within a *cis*-regulatory DNA sequence element (RegulomeDB bottom). (**c**) Presumptive disease risk model at rs208908. This variant, located upstream of *CXADR*, may affect lung cancer risk through gene expression regulation. The numbers indicate MAF for rs208908 in cases/controls between male and female population.

Further functional annotation analysis inferred rs208908 as a TBFS with probability of 0.92. On the basis of information from existing TFBS, an 8-bp TBFS motif containing rs208908 was predicted and the minor allele ‘A’ at rs208908 is predicted to increase the binding of TF of Glucocorticoid Receptor-β and YY1 (Fig. 3b). Both of these TFs have been reported to be involved in cancer development and YY1 is a dual function TF and has been implicated as a major driver of many cancers including lung cancer (28,29). DNase-seq analysis also identified a *cis*-regulatory region including rs208908 variant in human cells. These results, combined with eQTL analysis, suggested rs208908 regulated *CXADR* gene expression by interacting with cellular factors such as TFs that function through recognition of conserved sequence motif located upstream of gene coding sequence. In 2018, a large-scale GWAS including ~ 370 000 individuals with European ancestry showed that *CXADR* gene was associated with lung function (Forced Expiratory Volume/Forced Vital Capacity (FEV1/FVCratio)) (33). These multiple lines of evidence, from the results from our integrated study to that from previous reports, suggested a disease model for SNP rs208908 with sexual disparity in lung cancer risk. SNP rs208908 has different MAF between women (0.030) and men (0.025). This difference combined with differentiated gene expression regulation mechanism between men and women, leads to different gene expression patterns between these groups resulting in different risk between male and female groups (Fig. 3c).

A remarkable number of genes have been identified to be differentially expressed between male and females in one or more human tissues including lung (34). In addition, sex-biased regulatory targeting patterns have been found for various TFs in human (35). In our study, rs208908 was found to have differential risk effect for lung cancer between men and women, with different MAF between sexes (0.025 in women and 0.032 in men) (Supplementary Material, Table S7). And MAF was 0.023/0.028 between cases/controls in women and 0.033/0.031 in men, indicating more imbalance of allele frequency between lung cancer cases and controls in female group. Similar findings were also found for rs17662871 and rs79942605. We further compared the MAF for the variants between men and women in controls only, to exclude the selection bias for the cases in the data, and the *P*-value for rs17662871 was 1.88×10^−2^ and 0.088 for rs208908 which was around the border line statistical significance level. This sex-specific allelic frequency suggested a sexually antagonistic selection, a selection can occur when both sexes have different fitness optima for a trait, leading to genetic variations between male and female in a population (36,37). For example, the difference in allele frequency for rs208908, as a key variant in a TF binding site, may cause differential regulation of TFs between sexes, and then differential CXADR gene expression and different lung cancer risk between men and women.

For the other two variants, rs17662871 (close to *CA10*) and rs79942605 (within *MACROD2*), very few supporting variants were identified nearby, which is not unusual for variants with low allele frequency. The low allele frequency also makes eQTL analysis challenging. rs79942605 was not available in GTEx and there were only a few samples with homogenous minor-allele genotype at rs17662871 in GTEx genotype data, which limited our ability to investigate the gene expression pattern across different genotype groups. Both *CA10* and *MACROD2* were reported to be associated with smoking behavior and age at menarche in Caucasian (38,39). Controversial results have been reported for association between age at menarche and lung cancer risk (40–42). Our study detected an effect for lung cancer in only women group in these two regions, especially rs79942650 from *MACROD2* gene which has a risk effect in only smoking women, suggesting that sex plays an important role in lung cancer susceptibility and may interact with smoking behavior in cancer risk.

In summary, we conducted a large-scale gene–sex interaction scanning in lung cancer and we identified three significant variants with different risk effects on the basis of sex. Our study is one of the first studies to examine sex disparity in lung cancer development and our results provided insights about the genetic and molecular mechanism underlying the differences in lung cancer susceptibility between men and women. Our study is one of the largest scanning for gene–sex interaction in lung cancer in people with European ancestry. The three novel variants identified in our study all have MAF < 0.05. In our previous study to identify cross-ancestry loci contributing to lung cancer using multi-population genome-wide meta-analysis of 61 047cases and 947 237 controls, we identified five novel loci including three rare variants (MAF < 0.05) (31). These results suggest some variants still remain undetected in lung cancer, including those with low allele frequency requiring a larger sample size for more effective methods for detection.

## Material and Methods

### Materials

The imputed genotypes (reference panel: HRC r1.1) from 46 783 individuals with European ancestry, with lung cancer phenotype, smoking (ever vs. never smokers) and sex information, in the INTEGRAL-ILCCO lung cancer consortium were analyzed in this study (31). The subjects came from nine independent studies: the OncoArray Consortium Lung Study (OncoArray), including 16 845 lung cancer cases and 13 057 controls, was used as the discovery dataset (32,43). Individuals from another eight smaller independent studies: Affymetrix Axiom Array Study, the Genetic Epidemiology of Lung Cancer Consortium, the Environment and Genetics in Lung cancer Etiology study, the International Agency for Research on Cancer, MD Anderson Cancer Center Study, NCI Lung Cancer and Smoking Phenotypes in African-American Cases and Controls (NCI), the Prostate, Lung, Colorectal and Ovarian Cancer Screening Trial and Samuel Lunenfeld Research Institute Study were combined together and used as replication dataset including 7378 lung cancer cases and 9503 controls (Table 1, Supplementary Material, Table S1) (44–47). There were 12 084 women and 17 818 men in discovery dataset; and 6366 women and 10 515 men in replication dataset. About 60.4% of the lung cancer patients were male in the discovery study compared with 64.0% in replication study; 81.6% of individuals in both discovery and validation studies were ever smokers. Stringent quality controls were applied on SNPs and SNPs selected for the analysis had to qualify by two criteria: (1) had imputation information score ≥ 0.7 in discovery data; (2) had information score > 0.2 in each of the eight studies and sample-size weighted information score ≥ 0.7 in replication data. About ~ 20 000 000, out of 39 000 000 imputed SNPs, with information score ≥ 0.7 were analyzed in discovery study and ~ 10 000 000 SNPs were analyzed in replication study (Fig. 1). About 193 050 markers, common to the 9 studies and with linkage disequilibrium r^2^ value less than or equal to 0.5, were selected to calculate principal components in PLINK. Detailed information about data collection, genotype imputation and quality control procedures can be found from our earlier publication (29).

**Table 2:** Significant variants in gene–sex interaction analysis evaluated using Firth regression method

				Case-only								Case–control		
				Discovery		Replication		Meta-analysis				Combined data^a^		Score
SNP	rsID	MAF	GENE	OR	*P*	OR	*P*	*P*	OR	Q	I	OR	*P*	
17:49639139:G:A	rs17662871	0.049	CA10 distal	0.69	1.21××10^−7^	0.80	7.03×10^−2^	4.29×10^−8^	0.71	0.27	17.10	0.78	4.06×10^−3^	0.72
20:15644218:T:C	rs79942605	0.012	MACROD2	2.37	9.05×10^−8^	1.67	6.34×10^−2^	2.81×10^−8^	2.17	0.28	14.83	1.68	1.64×10^−2^	0.82
21:18785818:G:A	rs208908	0.039	CXADR distal	0.71	2.84×10^−6^	0.67	4.49×10^−3^	4.54×10^−8^	0.70	0.76	0.00	0.80	1.74×10^−2^	0.85

The results from analysis using Firth logistic regression method were reported for the three variants. Infor. Q indicated *P*-values for Cochrane’s Q statistic; I indicated I^2^ heterogeneity index (0–100); Score indicated the imputation quality score for the variants.

^a^Discovery and replication combined data were used for case–control validation analysis.

**Table 3:** Functional annotation of candidate SNPs from significant regions

SNP	ID	case-only	CADD	RegulomeDB	
POS	SNP	*P*	PHRED^a^	Function annotation	Probability^b^
17:49639139:G:A	rs17662871	4.29×10^−8^	2.534	TF binding or DNase peak	0.49
20:15642704:G:A	rs76314075	1.33×10^−7^	1.445	Other	0.18
20:15644218:T:C	rs79942605	2.81×10^−8^	8.228	TF binding or DNase peak	0.04
21:18776825:C:T	rs208900	5.87×10^−6^	0.668	TF binding or DNase peak	0.13
21:18779654:G:A	rs184089	5.79×10^−6^	1.066	Motif hit	0.48
21:18783833:G:A	rs11088636	4.24×10^−6^	0.039	Other	0.18
21:18784296:T:C	rs423598	4.78×10^−6^	2.243	TF binding or DNase peak	0.13
21:18785818:G:A	**rs208908**	**4.54×10** ^**−8**^	**5.773**	**TF binding or DNase peak**	**0.92**
21:18787462:T:C	rs208914	5.01×10^−6^	2.944	TF binding + DNase peak	0.61
21:18787572:T:C	rs9637031	1.34×10^−7^	4.018	TF binding + DNase peak	0.61
21:18787948:A:G	rs6517771	1.76×10^−6^	5.095	Motif hit	0.34
21:18788445:A:C	rs1389157	1.30×10^−6^	6.257	TF binding or DNase peak	0.03

SNPs with case-only joint *P*-value <1×10^−5^ were selected for annotation analysis. eQTL displayed the information from GTEx Portal. The annotation information for rs208908 was bolded.

^a^Scaled CADD score by expressing the rank in order of magnitude terms.

^b^RegulomeDB probability score is ranging from 0 to 1, with 1 being most likely to be a regulatory variant.

### Statistical methods for GWGI

Following the two-step analysis strategy, case-only analysis was first performed between SNP dosage (additive model) and sex (male and female were coded as 1 and 2, respectively) phenotype using lung cancer patients from discovery study (*n* = 16 845) and replication study (*n* = 7378) (case-only model, S denotes smoking status). Fixed-effect meta-analysis was conducted to combine information from both studies. Variants with case-only joint *P*-value <5×10^−8^ and joint *P*-values more significant than those from either study were selected for further validation using case–control analysis. All the samples in discovery and replication study including 24 223 lung cancer patients and 22 560 controls were applied in case–control analysis (Case–control model, D denotes disease status). The SNPs with case–control gene–sex interaction *P*-value < 0.05 were reported as significant findings. For the significant variants with low allele frequency (MAF < 0.05), we further validated the signals using firth logistic regression, a method designed for rare variants association test to reduce small-sample bias in regular logistic regression (Fig. 1) (48). The statistical analysis was adjusted for smoking status (ever and never smokers) and the first five principal components. The analysis was conducted in overall lung cancer as well as adenocarcinoma (No. Cases = 9630) and squamous lung cancer (No. Cases = 6019).

$$\begin{array}{l} \mathrm{Logit}\left(\mathrm{D}\right)\kern-1pt\sim{\beta}_0+{\beta}_1\ast \mathrm{SNP}+{\beta}_2\ast \mathrm{sex}+{\beta}_3\ast \mathrm{SNP}\ast \mathrm{sex}\\+\varepsilon\ \left(\mathrm{case}-\mathrm{control}\ \mathrm{model}\right)\end{array} $$

$$ \mathrm{Logit}\left(\mathrm{S}\right)\sim{\beta}_0+{\beta}_1\ast \mathrm{SNP}+\varepsilon\ \left(\mathrm{case}-\mathrm{only}\ \mathrm{model}\right) $$

### eQTL analysis

Genotype dosage and gene expression rpkm data from lung tissue were downloaded from GTEx (phs000424.GTEx.v7.p2). There were 377 individuals with European ancestry available for the analysis, including 244 men and 133 women. Average rpkm for the gene was used if there were duplicated samples. Individuals with rpkm < 0.25 were removed from the analysis. For imputed variants, we applied a best guess algorithm to assign most likely genotypes to each individual to transform the dosage into genotype. For variants with MAF ≥ 0.1, we adopted an additive model: dosage values ≤0.2 were coded as 0; dosage between 0.4 and 0.6 were coded as 1 and dosage ≥ 0.8 was coded as 2. For variants with MAF < 0.1, we adopted a dominant model: dosage values≤0.4 were coded as 0 (non-carrier group with no risk alleles) and dosage ≥ 0.6 was coded as 1 (carrier group with at least 1 risk allele). For each candidate SNP, the boxplot of log2(rpkm) across different genotype was displayed for all the individuals, men and women groups. Student’s test was performed to compare the mean log2(rpkm) across different genotype groups and general linear regression was conducted to test SNP–sex interaction in gene expression prediction.

$$\begin{array} \mathrm{Log}2\left(\mathrm{rpkm}\right)\sim{\beta}_0+{\beta}_1\ast \mathrm{SNP}+{\beta}_2\ast \mathrm{sex}+{\beta}_3\ast \mathrm{SNP}\\\ast \mathrm{sex} +\varepsilon\ \left(\mathrm{general}\ \mathrm{linear}\ \mathrm{model}\right) \end{array}$$

Gene–sex interaction and eQTL analyses were conducted using program R-4.0.2. R package logistf 1.23 was applied for Firth logistic regression analysis. PLINK 1.07 was used for meta-analysis.

### Functional annotation analysis

Two public functional annotation tools, CADD and RegulomeDB were applied for functional inference. CADD was designed to predict functional variants by integrating diverse information from wide range of function categories (25). It computed a score inferring the functional ranking of the variants which is helpful for fine mapping. RegulomeDB is a tool designed to predict regulatory DNA elements in the human genome on the basis of information from Gene Expression Omnibus, ENCODE and public literature (26). It adopted score of unified regulatory features model, trained on data from massively parallel reporter assays, to predict the functional variants in enhancer and promoter elements. RegulomeDB provides a probability score for the variant being TFBS, promotor or DNase hypersensitivity, etc. PROMO, an online tool to perform computational-based searches for gene expression regulatory sequence motif on the basis of TFBS database, was used to search for the functional motif with sequence containing the candidate variants as input (27).

## Supplementary Material

Gene-sex_manuscript_Appendix_01112022_ddac030

## Materials & Correspondence

Correspondence should be addressed to Yafang Li. Material requests should be addressed to Christopher I. Amos (chrisa@bcm.edu).
