# Supplementary material for: Genome-wide interaction analysis identified low-frequency variants with sex disparity in lung cancer risk
Source: Hum Mol Genet. 2022 Feb 9;31(16):2831–43. doi: 10.1093/hmg/ddac030 (PMC9402242; doi:10.1093/hmg/ddac030)
Supplement: Gene-sex_manuscript_Appendix_01112022_ddac030 [file gene-sex_manuscript_appendix_01112022_ddac030.docx]

**Supplementary**

Table S1. European sample distribution in the study.

|  | **Discovery** | **Replication** | | | | | | | |
| --- | --- | --- | --- | --- | --- | --- | --- | --- | --- |
| **Study** | **ONCO** | **Affy** | **GELCC** | **EAGLE** | **IARC** | **MDACC** | **NCI** | **PLCO** | **SLRI** |
| Case/control | 16845/13057 | 1346/2575 | 724/767 | 1908/1967 | 1825/2420 | 1145/1128 | 5/13 | 159/241 | 266/392 |
| Total | 29902 | 3921 | 1491 | 3875 | 4245 | 2273 | 18 | 400 | 658 |

The imputed genotypes (reference panel: HRC r1.1) from 46,783 individuals from INTEGRAL (Integrative Analysis of Lung Cancer Etiology and Risk)-ILCCO (International Lung Cancer Consortium) lung cancer consortium were analyzed in this study. The subjects came from 9 independent studies: the OncoArray Consortium Lung Study (OncoArray), including 16,845 lung cancer cases and 13,057 controls, was used as the discovery. Individuals from another 8 smaller independent studies: Affymetrix Axiom Array Study (AFFY), the Genetic Epidemiology of Lung Cancer (GELCC) Consortium, the Environment and Genetics in Lung cancer Etiology study (EAGLE), the International Agency for Research on Cancer (IARC), MD Anderson Cancer Center Study (MDACC), NCI Lung Cancer and Smoking Phenotypes in African-American Cases and Controls (NCI), the Prostate, Lung, Colorectal and Ovarian Cancer Screening Trial (PLCO), and Samuel Lunenfeld Research Institute Study (SLRI), were combined together and used as replication dataset including 7,378 lung cancer cases and 9,503 controls.

Table s2. Significant variants identified in genome-wide gene-sex interaction analysis using regular linear regression method.

|  |  |  |  | Case-only | | | | | | | | Case-control | |  |
| --- | --- | --- | --- | --- | --- | --- | --- | --- | --- | --- | --- | --- | --- | --- |
|  |  |  |  | Discovery | | Replication | | Meta-analysis | | | | Combined | | Score |
| SNP | rsID | MAF | GENE | OR | P | OR | P | P | OR | Q | I | OR | P |  |
| 17:49639139:G:A | rs17662871 | 0.049 | CA10 distal | 0.69 | 1.13E-07 | 0.80 | 7.03E-02 | 3.83E-08 | 0.71 | 0.28 | 15.43 | 0.78 | 4.02E-03 | 0.72 |
| 20:15644218:T:C | rs79942605 | 0.012 | MACROD2 | 2.38 | 8.72E-08 | 1.66 | 6.72E-02 | 2.92E-08 | 2.17 | 0.27 | 18.45 | 1.68 | 1.58E-02 | 0.82 |
| 21:18785818:G:A | rs208908 | 0.039 | CXADR distal | 0.71 | 2.67E-06 | 0.67 | 4.11E-03 | 3.97E-08 | 0.70 | 0.74 | 0.00 | 0.80 | 1.72E-02 | 0.85 |
| 8:47800487:G:A | rs75989684 | 0.004 | Intergenic^1^ | 0.10 | 3.86E-09 | 0.26 | 8.27E-02 | 1.55E-09 | 0.12 | 0.27 | 18.86 | 0.65 | 2.56E-01 | 0.75 |
| 8:47777829:A:G | rs141595931 | 0.004 | Intergenic^1^ | 0.13 | 9.31E-09 | 0.29 | 1.00E-01 | 3.59E-09 | 0.15 | 0.36 | 0 | 0.75 | 4.55E-01 | 0.75 |
| 8:47776577:A:G | rs13845564 | 0.003 | Intergenic^1^ | 0.08 | 2.95E-08 | 0.17 | 9.53E-02 | 8.76E-09 | 0.09 | 0.52 | 0 | 0.59 | 2.55E-01 | 0.73 |
| 8:47749801:T:G | rs74608521 | 0.004 | Intergenic^2^ | 0.12 | 2.89E-09 | 0.32 | 1.20E-01 | 1.84E-09 | 0.14 | 0.22 | 34.94 | 0.73 | 3.93E-01 | 0.75 |
| 16:11981487:G:A | rs3752426 | 0.309 | GSPT1 | 0.87 | 7.18E-08 | 0.93 | 1.11E-01 | 4.51E-08 | 0.89 | 0.20 | 39.00 | 0.97 | 3.29E-01 | 0.94 |

8 SNPs were significant in case-only analysis (joint p value < 5x10^-8^) and 3 of them were significant in further case-control evaluation (p value < 0.05) which were highlighted in bold. The three significant variants were further analyzed using Firth logistic regression and the results were provided in Table 2. Q indicated p-value for Cochrane's Q statistic; I indicated I^2 heterogeneity index (0-100); Score indicated the sample-size weighted imputation quality score for the variants. Intergenic 1: between LINC00293 and LOC100287846; intergenic 2: between ASNSP1 and LINC00293.

Table S3. Top 10 variants in case-only analysis in lung cancer subtypes.

|  | Discovery | | Replication | | Meta-analysis | | | |  |
| --- | --- | --- | --- | --- | --- | --- | --- | --- | --- |
| SNP | OR | P | OR | P | P | OR | Q | I | Gene |
| Lung Adenocarcinoma | | | | | | | | | |
| 1:96306787:A:G | 0.80 | 3.18E-09 | 0.95 | 4.87E-01 | 2.66E-08 | 0.83 | 0.03 | 78.33 | LOC100996635(dist=325767),LOC102723661(dist=150837) |
| 7:130659519:A:G | 0.84 | 7.88E-07 | 0.92 | 2.04E-01 | 8.01E-07 | 0.86 | 0.20 | 39.13 | LINC-PINT |
| 8:72732114:T:C | 0.67 | 6.01E-07 | 0.88 | 3.35E-01 | 1.63E-06 | 0.72 | 0.09 | 64.98 | EYA1(dist=457647),MSC(dist=21663) |
| 8:72733148:C:T | 0.67 | 6.01E-07 | 0.88 | 3.35E-01 | 1.63E-06 | 0.72 | 0.09 | 64.98 | EYA1(dist=458681),MSC(dist=20629) |
| 8:72721701:G:A | 0.68 | 6.48E-07 | 0.88 | 3.29E-01 | 1.68E-06 | 0.72 | 0.10 | 64.09 | EYA1(dist=447234),MSC(dist=32076) |
| 8:72723843:C:T | 0.68 | 6.46E-07 | 0.88 | 3.27E-01 | 1.72E-06 | 0.72 | 0.09 | 64.84 | EYA1(dist=449376),MSC(dist=29934) |
| 8:72723961:A:C | 0.68 | 6.33E-07 | 0.88 | 3.30E-01 | 1.72E-06 | 0.72 | 0.09 | 65.20 | EYA1(dist=449494),MSC(dist=29816) |
| 8:72731248:A:G | 0.68 | 7.10E-07 | 0.87 | 3.17E-01 | 1.73E-06 | 0.72 | 0.10 | 63.24 | EYA1(dist=456781),MSC(dist=22529) |
| 8:72723596:G:C | 0.68 | 6.60E-07 | 0.88 | 3.26E-01 | 1.74E-06 | 0.72 | 0.09 | 64.66 | EYA1(dist=449129),MSC(dist=30181) |
| 8:72723598:G:A | 0.68 | 6.60E-07 | 0.88 | 3.27E-01 | 1.75E-06 | 0.72 | 0.09 | 64.71 | EYA1(dist=449131),MSC(dist=30179) |
| Squamous lung cancer | | | | | | | | | |
| 16:70776998:C:T | 2.43 | 2.65E-06 | 2.10 | 5.10E-02 | 3.89E-07 | 2.36 | 0.73 | 0.00 | VAC14 |
| 5:46173866:C:G | 3.81 | 2.55E-06 | 2.24 | 7.02E-02 | 7.89E-07 | 3.26 | 0.31 | 2.25 | HCN1(dist=477646),NONE(dist=NONE) |
| 6:163806162:A:G | 2.25 | 5.22E-06 | 1.76 | 5.72E-02 | 1.03E-06 | 2.11 | 0.48 | 0.00 | DKFZp451B082(dist=38097),CAHM(dist=27935) |
| 5:46377489:T:G | 3.56 | 5.04E-06 | 2.28 | 5.76E-02 | 1.14E-06 | 3.12 | 0.39 | 0.00 | HCN1(dist=681269),NONE(dist=NONE) |
| 5:46027251:T:A | 3.59 | 8.27E-06 | 2.52 | 4.43E-02 | 1.25E-06 | 3.25 | 0.52 | 0.00 | HCN1(dist=331031),NONE(dist=NONE) |
| 5:45941196:C:T | 3.53 | 7.58E-06 | 2.39 | 4.77E-02 | 1.31E-06 | 3.15 | 0.46 | 0.00 | HCN1(dist=244976),NONE(dist=NONE) |
| 5:45487089:T:C | 3.96 | 7.66E-06 | 2.45 | 4.54E-02 | 1.42E-06 | 3.40 | 0.38 | 0.00 | HCN1 |
| 5:45951034:A:T | 3.50 | 7.43E-06 | 2.32 | 5.38E-02 | 1.48E-06 | 3.11 | 0.43 | 0.00 | HCN1(dist=254814),NONE(dist=NONE) |
| 5:45950172:C:T | 3.49 | 8.94E-06 | 2.35 | 4.99E-02 | 1.63E-06 | 3.10 | 0.45 | 0.00 | HCN1(dist=253952),NONE(dist=NONE) |
| 5:46365404:C:A | 3.53 | 7.32E-06 | 2.17 | 7.41E-02 | 2.19E-06 | 3.06 | 0.35 | 0.00 | HCN1(dist=669184),NONE(dist=NONE) |

The case-only p values from discovery and replication study as well as joint analysis were reported for top 10 variants in adenocarcinoma and squamous lung cancer subtypes.

Table S4. General linear regression between rs208908 and CXADR gene expression (log2rpkm).

|  | **Estimate** | **SD** | **t value** | **Pr(>F)** |  |
| --- | --- | --- | --- | --- | --- |
| **Without interaction** | | | | | |
| Sex | -0.14 | 0.05 | -2.97 | 3.15x10^-3^ | ** |
| geno | -0.04 | 0.10 | -0.37 | 0.71 |  |
| **With interaction term** | | | | | |
| Sex | -0.18 | 0.05 | -3.57 | 4.05x10^-4^ | *** |
| geno | -0.86 | 0.30 | -2.87 | 4.32x10^-3^ | ** |
| Sex:geno | 0.57 | 0.19 | 2.91 | 3.85x10^-3^ | ** |

Figure S5. Comparison of the results between case-only (regular logistic regression) and firth test.

Table S6. The sex-specific effects for 12 reported significant variants identified in overall lung cancer in European population.

|  |  |  |  | Female | | Male | | Male+Female | | Case-only |
| --- | --- | --- | --- | --- | --- | --- | --- | --- | --- | --- |
| SNP | Cytoband | Location | Nearest Gene | OR | P | OR | P | OR | P |  |
|  |  |  | **Overall Lung Cancer** |  |  |  |  |  |  |  |
| rs9865715 | 3p22.1 | exonic | CYP8B1 | 1.77 | 5.42E-08 | 1.49 | 2.38E-04 | 1.65 | 3.74E-11 | 5.65E-01 |
| rs12203592 | 6p25.3 | intronic | IRF4 | 1.10 | 2.82E-04 | 1.12 | 5.58E-05 | 1.12 | 7.52E-09 | 2.91E-03 |
| rs17534632 | 6q21 | intronic | PPIL6 | 1.10 | 6.55E-06 | 1.10 | 3.12E-04 | 1.10 | 3.73E-09 | 6.35E-02 |
| rs34102154 | 6p21.32 | intergenic | HLA-DRB1(dist=14493), HLA-DQA1(dist=33077) | 0.91 | 2.08E-05 | 0.88 | 2.29E-06 | 0.90 | 1.68E-09 | 8.07E-02 |
| rs2413932 | 15q21.1 | intergenic | SECISBP2L(dist=44721), COPS2 (dist=33990) | 1.09 | 4.31E-06 | 1.10 | 1.72E-05 | 1.09 | 1.65E-10 | 1.96E-01 |
| rs34517439 | 1p31.1 | intronic | DNAJB4 | 1.14 | 3.59E-06 | 1.17 | 1.63E-06 | 1.16 | 5.03E-12 | 9.88E-01 |
| rs2853677 | 5p15.33 | intronic | TERT | 0.90 | 5.93E-11 | 0.82 | 1.51E-22 | 0.86 | 3.11E-29 | 1.60E-02 |
| rs72477506 | 8p21.2 | intronic | EPHX2 | 0.83 | 5.36E-07 | 0.83 | 2.50E-05 | 0.83 | 5.27E-11 | 3.11E-01 |
| rs7850447 | 9p21.3 | intergenic | MIR31HG (dist=204051), MTAP (dist=38887) | 1.08 | 7.40E-03 | 1.18 | 2.39E-07 | 1.12 | 4.20E-08 | 9.94E-04 |
| rs71658797 | 1p31.1 | intronic | AK5 | 1.16 | 3.09E-06 | 1.14 | 3.24E-06 | 1.15 | 1.25E-11 | 9.17E-01 |
| rs11780471 | 8p21.2 | intergenic | CHRNA2(dist=7906), EPHX2 (dist=3800) | 0.84 | 2.37E-05 | 0.86 | 1.03E-05 | 0.85 | 9.86E-10 | 1.52E-01 |
| rs11571833 | 13q13.1 | exonic | BRCA2 | 1.54 | 6.42E-05 | 1.60 | 1.85E-07 | 1.57 | 5.91E-11 | 7.69E-01 |
| rs55781567 | 15q25.1 | UTR5 | CHRNA5(NM_001307945:c.-76C>G,NM_000745:c.-76C>G) | 1.25 | 1.31E-28 | 1.25 | 5.20E-40 | 1.25 | 1.56E-66 | 9.76E-01 |
| rs56113850 | 19q13.2 | intronic | CYP2A6 | 1.06 | 8.11E-03 | 1.13 | 9.58E-13 | 1.10 | 1.49E-13 | 9.75E-02 |
|  |  |  | **Lung adenocarcinoma** |  |  |  |  |  |  |  |
| rs13080835 | 3q28 | intronic | TP63 | 0.87 | 1.49E-07 | 0.91 | 6.01E-05 | 0.89 | 3.86E-11 | 9.52E-01 |
| rs7705526 | 5p15.33 | intronic | TERT | 1.26 | 2.05E-15 | 1.20 | 1.86E-12 | 1.23 | 2.08E-26 | 9.16E-01 |
| rs4236709 | 8p12 | intronic | NRG1 | 0.88 | 1.32E-04 | 0.88 | 6.79E-06 | 0.88 | 5.68E-09 | 1.72E-01 |
| rs885518 | 9p21.3 | intronic | MTAP | 1.26 | 2.55E-08 | 1.16 | 5.90E-05 | 1.20 | 3.10E-11 | 1.73E-01 |
| rs11591710 | 10q24.33 | intergenic | STN1(dist=9587), SLK (dist=39311) | 1.17 | 4.00E-05 | 1.14 | 1.34E-04 | 1.16 | 9.26E-09 | 2.13E-01 |
| rs1056562 | 11q23.3 | UTR3 | MPZL2(NM_005797:c.*336A>G) | 0.90 | 8.39E-05 | 0.91 | 2.69E-04 | 0.91 | 3.69E-08 | 1.04E-01 |
| rs77468143 | 15q21.1 | intergenic | SECISBP2L(dist=37864), COPS2 (dist=40847) | 0.87 | 8.56E-06 | 0.85 | 5.40E-09 | 0.85 | 9.42E-14 | 9.35E-01 |
|  |  |  | **Squamous lung cancer** |  |  |  |  |  |  |  |
| rs17879961 | 22q12.1 | exonic | CHEK2 | 0.35 | 4.50E-02 | 0.24 | 2.10E-10 | 0.26 | 3.36E-11 | 5.22E-01 |

22 significant variants in lung cancer from European population were retrieved from previous studies^29-30^. The OR and P values from regular GWAS (Male+Female) as well as sex-specific GWAS were reported for the 12 variants. The joint case-only analysis p value was also reported for each variant.

Table S7. Minor allele frequency for three variants between men and women in cases vs. controls in individuals with European ancestry.

|  |  |  |  |  | MAF | | | MAF comparison between men and women | | |
| --- | --- | --- | --- | --- | --- | --- | --- | --- | --- | --- |
| SNP | rsID |  | OR | P | Cases+controls | Cases | Controls | P1 | P2 | P3 |
| 17:49639139:G:A | rs17662871 | Female | 0.86 | 1.44E-02 | 0.0354 | 0.0325 | 0.0383 | 8.98x10^-9^ | 6.05x10^-9^ | 1.88x10^-2^ |
|  |  | Male | 1.06 | 2.20E-01 | 0.0460 | 0.0472 | 0.0447 |  |  |  |
| 20:15644218:T:C | rs79942605 | Female | 1.52 | 4.76E-03 | 0.0060 | 0.0071 | 0.0049 | 2.04x10-1 | 4.42x10^-2^ | 7.56x10^-1^ |
|  |  | Male | 0.99 | 9.59E-01 | 0.0051 | 0.0050 | 0.0052 |  |  |  |
| 21:18785818:G:A | rs208908 | Female | 0.81 | 1.17E-03 | 0.0253 | 0.0232 | 0.0275 | 7.42x10^-6^ | 4.571x10^-6^ | 8.71x10^-2^ |
|  |  | Male | 1.03 | 5.90E-01 | 0.0323 | 0.0330 | 0.0314 |  |  |  |

P1, P2 and P3 indicate the p values from minor allele frequency test between men and women in cases+controls, cases, and controls, respectively.

Suppose the MAF in men and women are p1 and p2, respectively. The number of individuals in male group is n1 and n2 for female group. Then $\hat{p1}-\hat{p2}$ is approximately normally distributed with mean p1-p2, and variance $\sqrt{\frac{p1(1-p1)}{n1}+\frac{p2(1-p2)}{n2}}$ . Then Z statistics is computed by ${(p1-p2)}/{\sqrt{\frac{p1(1-p1)}{n1}+\frac{p2(1-p2)}{n2}}}$ following an approximately normal distribution.
